# Neighborhood Environment, Internet Use and Mental Distress among Older Adults: The Case of Shanghai, China

**DOI:** 10.3390/ijerph18073616

**Published:** 2021-03-31

**Authors:** Dan Ma, Hao Yuan

**Affiliations:** 1 Department of Sociology, Shanghai Administration Institute, Shanghai 200233, China; madansoc@163.com; 2 School of Sociology and Political Science, Shanghai University, Shanghai 200444, China

**Keywords:** mental distress, neighborhood environment, Internet use, moderating effect, older adults

## Abstract

As the Internet evolves in urban communities, its consequences on mental distress have drawn significant research attention. We examine the relationships of mental distress with neighborhood environment and Internet use among older adults, using data from a representative sample of 2036 adults aged older than 60 years in Shanghai, China. We assess mental health with a 10-item scale from the Symptom Checklist 90 and Internet use with a 4-item scale and obtain information of neighborhood environment from an online map platform. Results from multilevel models show that both neighborhood environment and Internet use are significantly related to mental distress. Moreover, a worse neighborhood environment may strengthen the correlation between Internet use and mental distress, indicating the strong moderating role of the neighborhood environment. Thus, promoting Internet use among elderly people might result in a reduction in the prevalence of mental distress in disadvantaged neighborhoods.

## 1. Introduction

Rapid economic development has accelerated the increase in Internet use in China. According to Tencent’s Mobile Internet Report for Elderly Users, the number of elderly Internet users accounts for only 20% of the elderly population, although it has increased by 130% since 2012 [1]. In the United States, however, more than half of people over 65 had access to the Internet in 2012, with 70% of them reporting surfing online almost every day [2]. The population of elderly Internet users in China has great growth potential as the Internet keeps developing. 

With increasing Internet use, research on the relationship between Internet use and mental health has been on the rise. People usually use the Internet for the purposes of obtaining news and information, online social contact and interaction, online shopping, and online entertainment. The Internet provides convenience for its users and also influences their mental status to some extent [3]. Brailovskaia and Margraf find that Internet users have better mental health than non-Internet users [4]. Muscanell and Guadagno reveal that the Internet and social networking software may help people establish and maintain broader, more heterogeneous social relationships and social networks [5], which are considered positively relative to mental health [6]. Wang and colleagues report that using social networking websites is positively related to mental well-being among college students in Southwestern China [7]. When they use the Internet, people often have a sense of belonging, which is beneficial to their mental health [8]. 

In particular, the Internet provides a cushion against hardships and disadvantages. People with disabilities more frequently participate in physical activities and sport and, as a result, have greater happiness and better mental health [9]. The experience of using the Internet can also reduce mental distress among people with COVID-19 in the United States and five European countries [10]. Moreover, the association between Internet use and mental health is stronger among poor older adults than the rich [11]. 

Baker and Algorta point out that the majority of studies have shown that the relationship between Internet use and mental health is complex, and it may intensify the separation of some groups from the real world, promote loneliness and alienation, and cause mental distress [12]. Using data from the 2013 to 2015 Gallup Panel Social Network Survey data, Shakya and Christakis find that using the Internet is harmful to mental health [13]. Online social networking and conspicuous consumption may also induce social comparisons and cause mental distress [14]. Some studies on Internet addiction among adolescents and young adults show that excessive dependence on the Internet can cause severe mental disorders [15,16,17]. It may also exacerbate the rebellion of some Internet users against mainstream social values, promote loneliness and alienation, and cause indifference and fear. Therefore, scholars also find many unignorably negative consequences of Internet use on mental health. When people see others having higher incomes or better lives on the Internet, they are likely to have mental disorders such as jealousy, frustration, or disappointment [18]. 

In recent years, more and more elderly people have started to use the Internet. Compared with young people, elderly people use the Internet mainly to contact acquaintances as well as acquire news and information [19]. They are also more willing to participate in various online entertainment and social activities [20,21,22]. These connections and activities reduce the likelihood of mental distress [23]. In particular, it helps disadvantaged elderly people go beyond physical, mental, and social restrictions and carry out an increasingly diverse array of tasks and social activities [24]. In addition, elderly people usually have gone through important life events and many stressful situations such as retirement and a decline in physical health. These negative events do not necessarily undermine their mental health [25]. In fact, they usually have lower levels of mental disorders, such as diagnosable anxiety disorders, mood disorders, impulse-control disorders, and substance use disorders, compared with younger adult groups [26]. Therefore, we assume that Internet use may be positively related to better mental health among the elderly group.

Two categories of neighborhood environmental factors are crucial to mental distress: structural factors and social processes [27]. Structural factors consist of neighborhood socioeconomic composition, build and service environments, and residential stability. Social processes include neighborhood disorder, social cohesion, ties with neighbors, perceived exposure to crime, drug use, and graffiti. A good neighborhood environment can provide well-equipped infrastructures, harmonious social interactions with neighbors, social isolation, and a high level of neighborhood satisfaction and, thus, reduce the risks of having mental health problems [28,29].

The neighborhood environment can not only directly improve mental health but also counteract the impact of negative life events. When the balance of daily life is threatened, elderly people in communities with better social cohesion are more likely to successfully cope with such threats than others [30]. Extensive research demonstrates this association specifically for the older population in that older adults usually spend more time in community lives and obtain more social support from neighbors [31]. The majority of previous studies are limited to the direct effect of neighborhood environmental factors and disregard the role of the Internet in neighborhood effects and the possible interaction effect between neighborhood environment and Internet use. 

In China, many cities have started urban regenerations and constructions of community infrastructures based on the Internet. A great number of Internet applications help older adults obtain information, videos, communication, and social support from the Internet. As the cost of computers and smartphones has dropped, more and more Chinese elderly adults are gaining basic access to the Internet. However, the digital divide, accompanying the diffusion of the Internet, indicates that certain populations of older people have substantially better opportunities to benefit from the Internet than others. Social structure and economic conditions may determine who can benefit more from the Internet [32]. The Internet has transformed and created new norms for neighborhood communication. Young netizens often neglect their friends and neighbors and integrate into remote online relationships, possibly at the expense of local contact [33]. However, elderly people usually pay more attention to what is happening in their immediate surroundings and are more likely to use the Internet to maintain contact with neighbors by adding Internet contact to telephone and face-to-face contact [34]. Thus, Internet use may be profitable for mental health among elderly Chinese people.

The neighborhood environment may determine who can benefit more from the Internet. Living in neighborhoods with more social capital is protective against mental distress [35]. In Shanghai, elderly people living in disadvantaged neighborhoods may more frequently communicate with their neighbors and enjoy higher levels of psychological benefits [30,36,37,38]. The Internet, hence, might be more helpful for elderly people in worse neighborhoods to go beyond disadvantaged situations and obtain social support and public services from their neighbors and communities. 

Based on the literature mentioned above, we hypothesize that neighborhood environment and Internet use are directly related to mental health, and neighborhood environment may reduce the correlation between Internet use and mental health. To test these hypotheses, we use a large-scale data set from the Shanghai Neighborhood Survey conducted in the city of Shanghai, China. This city is the most developed and largest metropolis in China, with a population of 24 million residents [39]. It has about 5 million elderly people aged over 60. Hence, this study could provide evidence and information for public policy aiming at promoting successful aging and improving the elderly’s mental health in the age of the Internet. 

## 2. Materials and Methods

### 2.1. Data

This study analyzed data from the Shanghai Urban Neighborhood Survey, conducted by the Data Science and Survey Center of Shanghai University in 2017 in Shanghai, China. The survey employed multistage probability proportional to size sampling (PPS) with implicit stratification to reduce the operational cost and increase representativeness [30]. It constructed a citywide representative sample of 8631 residents aged 15 or above, nested in 190 communities. The survey is intended to be a new resource for the scientific studies of physical, psychological, social, and family characteristics in the megacity of Shanghai, China. Professionally trained interviewers conducted face-to-face individual interviews, lasting approximately 1 h. This study calculated neighborhood interaction and sense of neighborhood at the neighborhood level based on the total sample and tests the multilevel regression models with the subsample of 2036 respondents aged between 60 and 79 years old from 179 neighborhoods. To capture the sociodemographic characteristics of the neighborhood, this study also derived information on points of interest, respectively, around these neighborhoods from the Baidu online map platform (Baidu, Beijing, China) using Python (Python Software Foundation, Beaverton, OR, USA) and ArcGIS (Esri, Eedlands, CA, USA). 

### 2.2. Measurement

#### 2.2.1. Dependent Variable

The dependent variable was mental distress, measured with a ten-item version of the Symptom Checklist-90 [40]. Respondents were asked: “how did you feel last week?” They could choose the appropriate answer for the ten listed ten: “I feel fear for no reason,” “I feel afraid,” “I feel headache and dizzy and I can’t pull myself together,” “I feel nervous,” “I often blame myself,” “I didn’t sleep well,” “I feel depressed,” “I feel useless,” “I feel it difficult to do anything,” and “I feel there is no hope in the future.” For each item, response categories were “never or seldom” (=0), “several times” (=1), “sometimes” (=2), and “all the time” (=3). Some studies use this checklist to screen for mental distress in the Chinese context [41,42]. Total scores range from 0 to 30, with a higher score indicating greater mental distress. Cronbach’s alpha is 0.84 for this sample.

#### 2.2.2. Independent Variables

This study included Internet use and several variables on neighborhood environment as independent variables. The corresponding question on Internet use was: “How often do you collect information or participate in activities listed below on the Internet?” The listed items are: “reading current news,” “collecting health-related information,” “using QQ, WeChat or other social network sites,” and “playing games, watching videos, listening to songs and other entertainment activities”. For each item, response categories are “never or seldom” (=0), “several times” (=1), “sometimes” (=2), and “all the time” (=3). This study applied Principal Component Analysis (PCA) to construct an indicator of Internet use, ranging from −0.81 to 2.19. PCA shows that these 4 indicators can be combined to construct a new variable, with its eigenvalue reaching 3.06 (other potential variables’ eigenvalues are all less than 1), and over 76% of the total variance of this variable can be explained by the 4 original indicators, and their factor loading is over 0.8.

This study examined the influence of neighborhood environment from Neighborhood Socio-Economic Status (NSES) and Neighborhood Interaction. NSES is a multicomponent scale constructed using PCA and data from the Baidu online map platform (the largest online map platform in China) with geospatial information on several kinds of points of interest. These components were selected to capture socioeconomic differentials across neighborhoods: the number of aging institutes, the number of public parks or gardens, the number of restaurants, and the number of hospitals and the number of sports places. These kinds of institutes or places are located within the circles with a radius of 1 km centered on the offices of the 179 neighborhood committees. It proves that this kind of information is highly correlated with neighborhood socioeconomic attributes and community convenience [43]. PCA at the neighborhood level reports that we can construct a new factor based on these 5 components, with its eigenvalue reaching 4.1 (other potential variables’ eigenvalues are all less than 1), and over 82% of the total variance of this variable can be explained by the 8 original indicators, and their factor loading is over 0.8. This variable ranges from −1.083 to 3.394, with a higher score indicating better NSES. 

Neighborhood Interaction (NI) is aggregated from an individual variable to the neighborhood level. The individual variable refers to the Frequency of Visiting Neighbors (FVN). The correspondent question on FVN is how often they visit or chat with their neighbors in the past year. Response categories were as follows: “Almost every day” (=8), “twice or three time a week” (=7), “once a week” (=6), “twice a month” (=5), “once a month” (=4), “once a season” (=3), “twice a year” (=2), “once a year” (=1), and “almost never” (=0). We calculated the mean of FVN in each community based on the sample of 7226 adults aged from 18 to 79. 

#### 2.2.3. Control Variables

This study controlled for a set of variables to rule out plausible alternative effects on mental distress, including age, gender, marriage, years of education, household income, housing tenure, Hukou type, Chinese Communist Party membership (CCPM), self-reported Household Social Class (HSC), and chronic disease. Chronic disease refers to the number of chronic diseases the respondents have. The list of chronic diseases includes asthma, rheumatoid arthritis, hypertension, diabetes, hyperlipidemia, heart diseases, glaucoma, stroke, tumor, hepatitis/gallstones, gastroenteritis, and senile dementia. This study used the total number of these 12 types of diseases as an indicator of chronic disease. 

Table 1 reports the definition of key variables and descriptive statistical analysis. Female is coded as 0 and male as 1, and the sex ratio is very close to 0.5. The year of education reaches 10, indicating that the majority of elderly people in Shanghai have not finished high school education. One-fifth of them are CCP members. Furthermore, 87% of elderly people are married. Although 86% of them live in their own houses or apartments and 92% of them are Shanghai local residents, most of them consider themselves lower class.

#### 2.2.4. Analysis Strategy

With regard to analysis methods, we estimated multilevel linear regression models with the Stata software package 16.0 (StataCorp, College Station, TX, USA) due to the two-level hierarchical nature of the data (2007 individuals nested in 179 neighborhoods) and the continuous variable of mental distress [44]. We centered all variables by subtracting their means.

Multicollinearity was first detected using the variance inflation factor (VIF). The results showed that the VIF of each predictor variable in the model was less than 2, meaning no serious multicollinearity. Then, the null model was constructed to estimate the contribution of different levels of variables in explaining the differences in individuals’ mental distress in different neighborhoods. 

Intraclass correlation coefficients (ICCs) were used to estimate the extent to which the neighborhood-level variables accounted for the total variances in mental distress scores. Variance reduction ratios indicated the extent to which the neighborhood-level variables interpreted the variance of mental distress in different neighborhoods and were used to evaluate the suitability of the neighborhood-level variables in the models. The results from the null model show that the ICC is equal to 7.792%, indicating that multilevel regression models are necessary for this study and the majority of the variation in mental distress is at the individual rather than the neighborhood level.

This study used a stepwise hierarchical regression analysis approach to analyze the correlations of Internet use and neighborhood environment with mental distress at older ages. Two interaction terms of Internet use with NI and NSES were included to examine whether Internet use differentially affects mental distress across neighborhoods. This study fits models via maximum likelihood and used robust estimation.

## 3. Results

### 3.1. Mental Distress and Individual-Level Variables

Table 2 shows the results of three models exploring the correlations of Internet use and neighborhood environment with mental distress. First, Model 1 only includes the individual-level control variables. Women report significantly higher levels of mental distress than men (coefficient = −1.012, *p* < 0.001). Year of education is negatively related to mental distress (coefficient = −0.114, *p* < 0.001). Respondents with CCPM have less mental distress (coefficient = −0.777, *p* < 0.001). The number of chronic diseases is positively associated with mental distress (coefficient = 0.9, *p* < 0.001). Marital status, household income, and *hukou* status are associated with mental distress at the significant level of 0.1. Age and housing tenure are not significant covariates with mental distress.

Model 2 exams the correlations of mental distress with FVN and Internet use at the individual level, after adjusting for individual socioeconomic covariates. The results show that Internet use is negatively associated with mental distress (coefficient = −0.0394, *p* < 0.001), indicating that respondents who more intensively use the Internet would have much lower levels of mental distress. The more frequently visit and chat with neighbors, the lower levels of mental distress the respondents would have (coefficient = −0.071, *p* < 0.05). 

### 3.2. Mental Health and Neighborhood Environment

We take into account the effect of neighborhood environment in Model 3. The results from Model 3 display that NSES is significantly correlated with mental distress (coefficient = −0.349, *p* < 0.05), whereas the effect of NI on mental distress is significant at the level of 0.1. A one-unit increase in NSES decreases the score of mental distress by 0.349 units, indicating that mental distress tends to be higher in the socioeconomically disadvantaged neighborhoods.

Table 3 reports the cross-level interaction effects of Internet use and neighborhood environment on mental distress. In Model 4, the coefficient of the interaction item between NSES and Internet use is significant (coefficient = 0.206, *p* < 0.05), indicating that the impact of Internet use on mental distress significantly changes as the neighborhood environment improves. In the most disadvantaged neighborhood (NSES = −1.083), a one-unit increase in Internet use decreases the mental distress score by 0.622 units (=−0.399 − 1.083 × 0.206). Such a change in Internet use will lead to a decrease in mental distress by 0.399 units in an average neighborhood (NSES = 0). However, it may cause an increase in mental distress by 0.3 units (=−0.399 + 3.394 × 0.206) in the most affluent neighborhood (NSES = 3.394). Model 3 also includes the random effect item of Internet use. Its coefficient is almost equal to 0, indicating that the random effect of Internet use is negligible in this multilevel regression model. To sum up, Internet use plays a more important role in determining elderly netizens’ mental distress in socioeconomically disadvantaged neighborhoods. 

In Model 5, the coefficient of the interaction item between NI and Internet use is insignificant (ß = −0.110, SE = 0.094, *p* > 0.1), indicating that NI does not substantially determine the impact of Internet use on mental distress. Model 6 includes these two interaction items. Its results are consistent with the results from Model 4 and Model 5. 

Table 4 reports the respective associations of four items of Internet use with mental distress across neighborhoods in multilevel regression models. In Model 6, the coefficient of the interaction item between NSES and reading online news is significant (ß = 0.139, SE = 0.051, *p* < 0.01), indicating that the neighborhood environment significantly moderates the negative impact of reading online news on mental distress. A one-unit increase in the intensity of reading online news decreases the mental distress score by 0.315 units (=−0.165 − 1.083 × 0.139) in the most disadvantaged neighborhood but increases mental distress by 0.307 units (=−0.165 + 3.394 × 0.139) in the most affluent neighborhood. In an average neighborhood (NSES = 0), such a change causes a decrease in mental distress by 0.165 units. The results from Model 8 display a similar interaction effect between neighborhood environment and online social networking on mental distress. Meanwhile, the intensity of playing games, watching videos, listening to songs, and other entertainment activities shows a consistent association with mental distress across neighborhoods. Obtaining online health information is almost insignificantly associated with mental distress.

## 4. Discussion

In recent years, many scholars have discussed the relationship between Internet use and mental health, especially the phenomenon of “Facebook Depression” [6]. Nowadays, in China, there is an increasing number of people using the Internet. Therefore, the relationship between Internet use and subjective well-being has gradually drawn more and more attention.

This study tends to expand the body of knowledge in this area by investigating, using the representative sample of 2007 elderly adults from Shanghai, whether and to what extent the association between mental distress and Internet use varies with neighborhood environment measured with NESE and NI. NSES well represents the socioeconomic conditions and life convenience around certain communities. The results show that Internet use and NSES, in general, are negatively related to mental distress, after controlling for socioeconomic covariates, consistent with the analyses in previous studies [37,38,45]. While the high frequency of visiting neighbors seems related to lower levels of mental distress, NI is not significantly associated with individuals’ mental distress. It suggests that community social capital may have an indirect effect on mental distress by increasing social interactions at the individual level.

We further examine the cross-level interactions between neighborhood environment and Internet use. NSES substantially moderates the relationship between Internet use and mental distress. Using the Internet more frequently leads to lower levels of mental distress in disadvantaged neighborhoods but higher in affluent neighborhoods. These relationships hold in the face of stringent sociodemographic variables and chronic diseases. It implies that the marginal utility of Internet use against mental distress declines as NSES goes better. When living in disadvantaged neighborhoods, elderly Shanghainese people may be more dependent on online networking and online platforms to obtain medical services, social connections, and social support, and thus, the protective effect of Internet use against mental distress is much stronger [19,20].

If the elderly can conveniently get such resources from their neighborhoods, the protective effect will decline, and its negative effect on mental health will show up. These results offer a new understanding of neighborhood characteristics and Internet use that might contribute to mental health. Elderly people living in disadvantaged neighborhoods experience higher levels of mental distress and depend more on the Internet to reduce the odds of having these mental health problems.

Further analyses demonstrate that both reading online news and chatting online are associated with lower levels of mental distress, and the associations are also conditioned by NSES. These two kinds of online activities may be the buffer of key factors for elderly people from many of the stressors in disadvantaged neighborhoods. For instance, searching and sharing discount online commodities and information may reduce living expenses and thus improve the mental health of poor elderly adults in these neighborhoods. The intensity of using online entertainment is strongly negatively associated with mental distress in any neighborhood, indicating that those living in affluent neighborhoods can also mentally benefit from online activities such as watching online videos and playing online games. Further studies are needed to empirically demonstrate which kinds of information and social communication are more important for reducing elderly people’s mental distress in these disadvantaged neighborhoods.

Several limitations of this study should be addressed. First, this study is based on data from the city of Shanghai, one of the most developed areas in China. Therefore, these findings may not be generalizable to the whole country, especially not to the undeveloped rural areas [28]. Second, the four items of Internet use may not fully reflect various dimensions of Internet use, especially the use of mobile Internet [46]. Third, the use of cross-sectional survey data makes it impossible to assess the causal relationships between Internet use and mental distress. For example, unobserved confounders may have resulted in biased estimates of Internet use and mental distress. Those with higher levels of mental distress may be less likely to participate in online activities or play online games with others. Fourth, NSES and NI may not have fully captured the environmental stressors or supportiveness. The two variables do not assess the quality, affordability, or utilization of infrastructures and services in these neighborhoods. For instance, we calculate the number of restaurants around each neighborhood but do not measure whether or how often elderly people have had diners in those restaurants. Therefore, further studies are needed to collect longitudinal data from representative samples of the whole country, distinguish the different effects of the Internet and smartphones, and include more indicators measuring neighborhood characteristics.

## 5. Conclusions

This study expands on the body of knowledge in this area by investigating, using the representative sample of 2007 elderly adults from Shanghai, whether and to what extent the association between mental distress and Internet use varies with neighborhood environment. The results from this study show that the mental distress of elderly Shanghainese people depends to a large extent on individual characteristics and for a small part of the neighborhood environment. For older adults, changes in mental distress are more a function of underlying individual-level social and aging processes than of the impact of the neighborhood environment. Internet use and FVN, in general, are significantly negatively associated with mental distress at the individual level. NSES may reduce the odds of mental distress at the neighborhood level, after controlling for the individual-level socioeconomic covariates. Moreover, the cross-level analyses reveal the inequalities in psychological distress by the intensity of Internet use, with increased risks for the elderly in economically deprived neighborhoods. Those living in advantaged neighborhoods are much less able to make use of the potential benefits of the Internet. In particular, subsequent analyses show that NSES significantly reduce the effects of reading online news and online social networking on mental distress but not for obtaining health information or attending online entertainment activities. Therefore, this study gives evidence that Internet use is of potential importance in the prevention of mental distress of the elderly in economically disadvantaged neighborhoods. This could have implications for the improvement of mental health among elderly people in the age of the Internet. Policymakers need to be aware of the conditioning effects of neighborhood environments when providing online services and applications for elderly people. They are, thus, urged to develop programs and services that help more elderly people better use the Internet and improve neighborhood conditions and the Internet infrastructures in disadvantaged neighborhoods. In addition, they should also develop programs to minimize the negative effect of the Internet on mental health and monitor the potential emergence of Internet addiction in affluent neighborhoods.

## Figures and Tables

**Table 1:** Definition of key variables and descriptive analysis (*n* = 2007).

Variable	Definition	Mean	S.D.
Mental distress	Sum of 10 items on mental distress	3.27	4.70
Internet use	The principal component factor of four items on using the Internet	0	1
NSES	The principal component factor of 5 items on the neighborhood socioeconomic status	0	0.97
NI	the mean of FVN in each community based on the sample of 7226 adults	4.80	1.08
FVN	the frequency of visiting or chatting with neighbors	5.47	3.19
Age	Aged from 60 to 79	66.91	5.06
Education	Years of education	10.01	3.38
Household income	Total household income in 2016, measured on a 11-point scale (under 7000 to 500,000 or over) with the median being “50,000 to 99,999”	6.06	1.89
Being married	Married = 1; unmarried = 0	0.87	0.34
Housing tenure	Yes = 1; No = 0	0.86	0.35
Hukou type	Local (=1) vs. nonlocal (=0)	0.92	0.27
Gender	Male = 1; Female = 0	0.49	0.50
CCPM	Yes = 1; No = 0	0.21	0.41
Chronic Diseases	Number of chronic diseases	2.01	1.43
HSC	Self-reported household social class measured on a 10 point scale (1 = lowest; 10 = highest)	3.25	1.60

NSES: Neighborhood Socio-Economic Status, NI: Neighborhood Interaction, FVN: Frequency of Visiting Neighbors, CCPM: Chinese Communist Party membership, HSC: self-reported Household Social Class.

**Table 2:** Multilevel models on mental distress, Internet use, and neighborhood environments.

Model Predictors	Model 1 (Only Control Variables)		Model 2 (Internet Use + FNV)		Model 3 (NSES + NI)	
	Coeff.	t	Coeff.	t	Coeff.	t
Individual-level variables						
Age	−0.015	−0.763	−0.028	−1.353	−0.028	−1.349
Gender (ref: female)	−1.012 ***	−4.685	−1.105 ***	−5.085	−1.165 ***	−5.266
Marital status (ref: nonmarried)	−0.657 ^†^	−1.822	−0.722 *	−2.010	−0.781 *	−2.189
Year of education	−0.114 ***	−3.358	−0.074 ^†^	−1.795	−0.036	−0.836
Household income	−0.116 ^†^	−1.750	−0.098	−1.471	−0.075	−1.124
House tenure Chronic diseases	−0.388	−1.164	−0.352	−1.073	−0.598 ^†^	−1.682
	0.900 ***	11.016	0.897 ***	11.258	0.903 ***	11.471
Hukou	−0.730 ^†^	−1.843	−0.690 ^†^	−1.755	−0.657 ^†^	−1.677
Social class	−0.429 ***	−6.232	−0.426 ***	−6.189	−0.403 ***	−5.730
CCPM	−0.777 ***	−3.999	−0.682 ***	−3.549	−0.709 ***	−3.570
Internet use			−0.394 ***	−3.345	−0.337 **	−2.896
FVN			−0.071 *	−2.258	−0.086 **	−2.731
Constant	1.421 ***	0.034	1.418 ***	0.034	1.418 ***	0.034
Neighborhood-level variables						
NSES					−0.349 *	−2.424
NI					0.223 ^†^	1.737
Random-effects Parameters						
Between-group variance	1.108	0.425	1.027	0.394	0.850	0.342
Within-group variance	17.136	1.172	17.030	1.162	17.034	1.172
ICC	6.071%		5.685%		4.751%	
AIC	11517		11504		11496	

Note: Coeff. = Coefficient; ICC refers to Intraclass correlation coefficient; AIC refers to akaike information criterion; ^†^ *p* < 0.10, * *p* < 0.05, ** *p* < 0.01, *** *p* < 0.001.

**Table 3:** The cross-level interaction models on mental distress, Internet use, and neighborhood environments.

Model Predictors	Model 4 (Internet Use* NSES)		Model 5 (Internet Use* NI)		Model 6 (Internet Use* NSES & Internet Use * NI)	
	Coeff.	t	Coeff.	t	Coeff.	t
Individual-level variables						
Age	−0.026	0.021	−0.028	0.021	−0.026	0.021
Gender (ref: female)	−1.165 ***	0.221	−1.154 ***	0.221	−1.159 ***	0.221
Marital status (ref: nonmarried)	−0.789 *	0.354	−0.777 *	0.357	−0.786 *	0.355
Year of education	−0.027	0.043	−0.036	0.043	−0.027	0.043
Household income	−0.065	0.067	−0.072	0.067	−0.064	0.067
House tenure	−0.624 ^†^	0.358	−0.613 ^†^	0.355	−0.632 ^†^	0.358
Chronic diseases	0.905 ***	0.078	0.902 ***	0.079	0.904 ***	0.079
Hukou	−0.680 ^†^	0.393	−0.662 ^†^	0.392	−0.682 ^†^	0.393
Social class	−0.409 ***	0.071	−0.403 ***	0.070	−0.409 ***	0.071
CCPM	−0.721 ***	0.198	−0.722 ***	0.199	−0.728 ***	0.199
FVN	−0.086 **	0.031	−0.084 **	0.032	−0.085 **	0.032
Internet use	−0.399 **	0.124	−0.373 **	0.117	−0.417 ***	0.122
Constant	1.418 ***	0.034	1.418 ***	0.034	1.418 ***	0.034
Neighborhood-level variables						
NSES	−0.398 **	0.149	−0.334 *	0.144	−0.385 *	0.151
NI	0.177	0.129	0.202 ^†^	0.120	0.167	0.122
Cross-level variables						
Internet use×NSES	0.206 *	0.089			0.193 *	0.093
Internet use×NI			−0.110	0.094	−0.068	0.097
Random-effects Parameters						
Between-group variance	0.802		0.825	0.340	0.791	0.329
Within-group variance	17.032		17.041	1.171	17.036	1.168
ICC	4.498%		4.620%		4.439%	
Aic	11497		11499		11498	

Note: Coeff. = Coefficient; ICC refers to Intraclass correlation coefficient; AIC refers to akaike information criterion; ^†^ *p* < 0.10, * *p* < 0.05, ** *p* < 0.01, *** *p* < 0.001.

**Table 4:** The cross-level interaction models on mental distress, Neighborhood Socio-Economic Status (NSES), and four items of Internet use.

Model Predictors	Model 7 (Online News)		Model 8 (Online Health Information)		Model 9 (Social Networking)		Model 10 (Online Entertainment)	
	Coeff.	t	Coeff.	t	Coeff.	t	Coeff.	t
Individual-level variables								
Age	−0.020	0.021	−0.021	0.020	−0.023	0.021	−0.028	0.020
Gender (0 = female)	−1.114 ***	0.220	−1.158 ***	0.220	−1.190 ***	0.223	−1.186 ***	0.222
Marital status (0 = nonmarried)	−0.760 *	0.354	−0.759 *	0.356	−0.795 *	0.356	−0.799 *	0.354
Year of education	−0.042	0.042	−0.052	0.042	−0.036	0.041	−0.039	0.039
Household income	−0.069	0.067	−0.075	0.068	−0.072	0.066	−0.070	0.066
House tenure	−0.638 ^†^	0.358	−0.641 ^†^	0.361	−0.638 ^†^	0.358	−0.607 ^†^	0.354
Chronic diseases	0.904 ***	0.079	0.905 ***	0.079	0.909 ***	0.079	0.903 ***	0.078
Hukou	−0.667 ^†^	0.392	−0.652 ^†^	0.394	−0.655 ^†^	0.392	−0.702 ^†^	0.395
Social class	−0.407 ***	0.070	−0.407 ***	0.071	−0.404 ***	0.070	−0.405 ***	0.070
CCPM	−0.746 ***	0.199	−0.735 ***	0.200	−0.741 ***	0.196	−0.727 ***	0.199
FVN	−0.087 **	0.032	−0.087 **	0.032	−0.086 **	0.032	−0.085 **	0.031
Online news	−0.165 *	0.068						
Online health news			−0.154 ^†^	0.081				
SNS					−0.207 **	0.074		
Online entertainment							−0.322 ***	0.069
Constant	1.418 ***	0.034	1.419 ***	0.034	1.418 ***	0.034	1.415 ***	0.034
Neighborhood-level variables								
NI	0.192	0.129	0.209	0.129	0.176	0.130	0.206	0.129
NSES	−0.421 **	0.147	−0.402 **	0.149	−0.416 **	0.147	−0.359 *	0.149
Cross-level variables								
Online news*NSES	0.139 **	0.051						
Online health news*NSES			0.091	0.066				
SNS*NSES					0.149 **	0.052		
Online entertainment*NSES							0.077	0.055
Random-effects parameters								
Between-group variance	0.809	0.331	0.829	0.339	0.823	0.336	0.860	0.340
Within-group variance	17.061	1.168	17.084	1.173	17.037	1.170	16.958	1.166
ICC	0.045		0.046		0.046		0.048	
AIC	11500		11505		11499		11492	

Note: Coeff. = Coefficient; ICC refers to Intraclass correlation coefficient; AIC refers to akaike information criterion; ^†^ *p* < 0.10, * *p* < 0.05, ** *p* < 0.01, *** *p* < 0.001.

## Data Availability

This study does not report any public data.

## References

[1] Tencent Company (2018). Mobile Internet Report for Elderly Users. https://www.sohu.com/a/231859559_300488.

[2] Zickuhr K., Madden M. (2012). Older Adults and Internet Use: For the First Time, Half of Adults Ages 65 and Older Are Online. http://pewinternet.org/~/media//Files/Reports/2012/PIP_Older_adults_and_internet_use.pdf.

[3] Davis R.A. (2001). A cognitive-behavioral model of pathological Internet use. Comput. Hum. Behav..

[4] Brailovskaia J., Margraf J. (2016). Comparing Facebook users and Facebook non-users: Relationship between personality traits and mental health variables—An exploratory study. PLoS ONE.

[5] Muscanell N., Guadagno R. (2012). Make new friends or keep the old: Gender and personality differences in social networking use. Comput. Hum. Behav..

[6] Johnston K., Tanner M., Lalla N., Kawalski D. (2013). Social capital: The benefit of Facebook ‘friends’. Behav. Inf. Technol..

[7] Wang J.L., Jackson L.A., Gaskin J., Wang H.Z. (2014). The effects of Social Networking Site (SNS) use on college students’ friendship and well-being. Comput. Hum. Behav..

[8] Nadkarni A., Hofmann S.G. (2012). Why do people use Facebook?. Personal. Individ. Differ..

[9] Duplaga M., Szulc K. (2019). The Association of Internet Use with Wellbeing, Mental Health and Health Behaviours of Persons with Disabilities. Int. J. Environ. Res. Public Health.

[10] Sigurvinsdottir R., Gylfason H.F. (2020). The Impact of COVID-19 on Mental Health: The Role of Locus on Control and Internet Use. Int. J. Environ. Res. Public Health.

[11] Yuan H. (2021). Internet use and mental health problems among older people in Shanghai, China: The moderating roles of chronic diseases and household income. Aging Ment. Health.

[12] Baker D., Algorta G. (2016). The relationship between online social networking and depression: A systematic review of quantitative studies. Cyberpsychol. Behav. Soc. Netw..

[13] Shakya H., Christakis N. (2017). Association of Facebook use with compromised well-being: A longitudinal study. Am. J. Epidemiol..

[14] Tandoc E., Ferrucci P., Duffy M. (2015). Facebook use, envy, and depression among college students: Is facebooking depressing?. Comput. Hum. Behav..

[15] Salicetia F. (2015). Internet addiction disorder. Soc. Behav. Sci..

[16] Wartberg L., Kammerl R. (2020). Empirical Relationships between Problematic Alcohol Use and a Problematic Use of Video Games, Social Media and the Internet and Their Associations to Mental Health in Adolescence. Int. J. Environ. Res. Public Health.

[17] Tan Y., Chen Y., Lu Y., Li L. (2016). Exploring Associations between Problematic Internet Use, Depressive Symptoms and Sleep Disturbance among Southern Chinese Adolescents. Int. J. Environ. Res. Public Health.

[18] Krasnova H., Wenninger H., Widjaja T., Bruxmann P. Envy on Facebook: A hidden threat to users’ life satisfaction?. Proceedings of the 11th International Conference on Wirtschaftsinformatik (WI2013).

[19] Choi N., DiNhto D., Eysenbach G. (2013). Internet use among older adults: Association with health needs, psychological capital and social capital. J. Med. Internet Res..

[20] Cresci M., Jarosz P., Templin T. (2012). Are health answers online for older adults?. Educ. Gerontol..

[21] Porter C., Donthu N. (2006). Using the technology acceptance model to explain how attitudes determine Internet usage: The role of perceived access barriers and demographics. J. Bus. Res..

[22] Hunsaker A., Hargittai E. (2018). A review of Internet use among older adults. New Med. Soc..

[23] Perkins E., LaMartin K. (2012). The Internet as social support for older carers of adults with intellectual disabilities. J. Policy Pract. Intellect. Disabil..

[24] Sum S., Mathews R., Hughes I. (2009). Participation of older adults in cyberspace: How Australian older adults use the Internet. Australas. J. Ageing.

[25] George L., Okun M., Landerman R. (1985). Age as a moderator of the determinants of life satisfaction. Res. Aging.

[26] Kessler R., Berglund P., Demler O., Jin R., Walters E. (2005). Lifetime prevalence and age-of-onset distributions of DSM-IV disorders in the National Comorbidity Survey Replication (NCS-R). Arch. Gen. Psychiatry.

[27] Mair C., Diez Roux A., Galea S. (2008). Are neighbourhood characteristics associated with depressive symptoms? A review of evidence. J. Epidemiol. Community Health.

[28] Wang Y., Chen Y., Shen H., Morrow-Howell N. (2018). Neighborhood and Depressive Symptoms: A Comparison of Rural and Urban Chinese Older Adults. Gerontologist.

[29] Tao Y., Yang J., Chai Y. (2020). The Anatomy of Health-Supportive Neighborhoods: A Multilevel Analysis of Built Environment, Perceived Disorder, Social Interaction and Mental Health in Beijing. Int. J. Environ. Res. Public Health.

[30] Miao J., Wu X., Sun X. (2019). Neighborhood, social cohesion, and the Elderly’s depression in Shanghai. Soc. Sci. Med..

[31] Glass T., Balfour J., Kawachi I., Berkman L. (2009). Neighborhoods, aging, and functional limitations. Neighborhoods and Health.

[32] Van Dijk J., Hacker K. (2003). The digital divide as a complex and dynamic phenomenon. Inf. Soc..

[33] Liu Q., Pan H., Wu Y. (2020). Migration Status, Internet Use, and Social Participation among Middle-Aged and Older Adults in China: Consequences for Depression. Int. J. Environ. Res. Public Health.

[34] Wellman B., Quan-Haase A., Witte J., Hampton K. (2001). Does the Internet Increase, Decrease, or Supplement Social Capital. Am. Behav. Sci..

[35] Hampton K., Wellman B. (2003). Neighboring in Netville: How the Internet Supports Community and Social Capital in a Wired Suburb. City Community.

[36] Nyqvist F., Forsman A., Giuntoli G., Cattan M. (2013). Social capital as a resource for mental well-being in older people: A systematic review. Aging Ment. Health.

[37] Qiu Y., Liu Y., Liu Y., Li Z. (2019). Exploring the Linkage between the Neighborhood Environment and Mental health in Guangzhou, China. Int. J. Environ. Res. Public Health.

[38] Dong H., Qin B. (2017). Exploring the link between neighborhood environment and mental wellbeing: A case study in Beijing, China. Landsc. Urban Plan..

[39] Shanghai Municipal Statistics Bureau (2017). 2017 Shanghai Statistical Yearbook.

[40] Derogatis L. (1977). SCL-90-R: Administration, Scoring and Procedures Manual.

[41] Xin S., Jiang W., Xin Z. (2019). Changes in Chinese nurses’ mental health during 1998–2016: A cross-temporal meta-analysis. Stress Health.

[42] Xin Z., Zhang M. (2009). Changes in Chinese middle school students’ mental health (1992~2005): A cross-temporal meta-analysis. Acta Psychol. Sin..

[43] Lei D., Rattia C., Zheng S. (2019). Predicting neighborhoods’ socioeconomic attributes using restaurant data. Proc. Natl. Acad. Sci. USA.

[44] Arcaya M.C., Tucker-Seeley R.D., Kim R., Schnake-Mahl A., So M., Subramanian S.V. (2016). Research on neighborhood effects on health in the United States: A systematic review of study characteristics. Soc. Sci. Med..

[45] Aneshensel C.S., Wight R.G., Miller-Martinez D., Botticello A.L., Karlmangla A.S., Seeman T.E. (2007). Urban neighborhoods and depressive symptoms among older adults. J. Gerontol. Soc. Sci..

[46] Višnjić A., Veličković V., Sokolović D., Stanković M., Mijatović K., Stojanović M., Milošević Z., Radulović O. (2018). Relationship between the Manner of Mobile Phone Use and Depression, Anxiety, and Stress in University Students. Int. J. Environ. Res. Public Health.

